# Federated Learning for Predictive Maintenance and Anomaly Detection Using Time Series Data Distribution Shifts in Manufacturing Processes

**DOI:** 10.3390/s23177331

**Published:** 2023-08-22

**Authors:** Jisu Ahn, Younjeong Lee, Namji Kim, Chanho Park, Jongpil Jeong

**Affiliations:** 1 Department of Smart Factory Convergence, Sungkyunkwan University, 2066 Seobu-ro, Jangan-gu, Suwon-si 16419, Gyeonggi-do, Republic of Korea; js3053@skku.edu (J.A.); ioioiiopop@g.skku.edu (Y.L.); namji@skku.edu (N.K.); chanho124@g.skku.edu (C.P.); 2 AI Research Center, Gfyhealth, 20 Pangyo-ro, Bundang-gu, Seongnam-si 13488, Gyeonggi-do, Republic of Korea

**Keywords:** federated learning, anomaly detection, time series, LSTM, data distribution

## Abstract

In the manufacturing process, equipment failure is directly related to productivity, so predictive maintenance plays a very important role. Industrial parks are distributed, and data heterogeneity exists among heterogeneous equipment, which makes predictive maintenance of equipment challenging. In this paper, we propose two main techniques to enable effective predictive maintenance in this environment. We propose a 1DCNN-Bilstm model for time series anomaly detection and predictive maintenance of manufacturing processes. The model combines a 1D convolutional neural network (1DCNN) and a bidirectional LSTM (Bilstm), which is effective in extracting features from time series data and detecting anomalies. In this paper, we combine a federated learning framework with these models to consider the distributional shifts of time series data and perform anomaly detection and predictive maintenance based on them. In this paper, we utilize the pump dataset to evaluate the performance of the combination of several federated learning frameworks and time series anomaly detection models. Experimental results show that the proposed framework achieves a test accuracy of 97.2%, which shows its potential to be utilized for real-world predictive maintenance in the future.

## 1. Introduction

Predictive maintenance (PdM) is the strategy to predict equipment failures and perform maintenance to prevent them before they occur [1]. This strategy is very important in the era of Industry 4.0 and smart manufacturing. By leveraging sensor readings, process parameters, and other operational characteristics, PdM can help reduce the number of unnecessary repairs while maximizing the lifespan of equipment by reducing the likelihood of unexpected failures [2]. This strategy reduces machine maintenance costs and maximizes machine uptime, leading to increased productivity. Since most manufacturing processes follow assembly line production, failure of a machine causes a domino effect. Therefore, it is important to avoid failure points within the assembly line. In order to make the most accurate and optimal predictions, it is essential to collect and analyze a large amount of relevant data within a reasonable amount of time [3,4].

Since industrial parks are spread across multiple locations, data collection in real time can be a problem that increases rapidly in scale. In traditional centralized cloud systems, data are often sent to a central server for processing. However, this approach has several problems.

- It increases the load on the central server. When a large amount of data from multiple locations is sent to a centralized server in real time, the data processing volume becomes very large and may exceed the processing power of the central server or cause network bottlenecks. This can result in delays or loss of data, which can reduce the performance of the real-time predictive maintenance system.
- Data security issues may arise. In a centralized system, all data are sent to a central server for processing, which can lead to data security and privacy issues. Critical manufacturing process data or confidential information can be exposed, and data leaks can occur if malicious actors are involved. This can lead to a decrease in competitiveness or even costly losses for the organization [5].

To address these challenges, it is useful to introduce intelligent edge computing systems for distributed data processing. Edge computing refers to the practice of processing data on edge devices or local servers close to the source of data generation. This allows data to be processed at the source and reduces the amount of data sent to a central server. Edge devices analyze data in real time and send only needed information to the central server, significantly reducing system load.

Federated Learning (FL) has emerged as a distributed, collaborative AI approach that can enable many intelligent IoT applications by enabling AI training on distributed IoT devices without the need to share data [6]. In an Edge–Cloud structure, each edge creates a model through individual training and only updates the parameters (weights, etc.) of each model per edge. Then, the cloud server creates a global model based on the edge models, solving the aforementioned problems [7]. However, in associative learning, the statistical heterogeneity of device data collected at each edge must be considered [8]. Statistical heterogeneity includes data heterogeneity between heterogeneous devices, different distributions of data collected over time from the same device, and stark differences in the class distribution of training data. Since training a model with heterogeneous data is likely to lead to model scatter, a structure is needed to address the problems arising from data heterogeneity.

In the literature, various studies have been conducted to improve the efficiency of fault prediction in edge IoT devices by building a global prediction model based on a local prediction model [9] and association learning for predictive maintenance and quality inspection in industrial applications [10]. However, there have been no studies on anomaly detection models and association learning that consider the distributional shifts of time series data collected from devices for predictive maintenance of manufacturing processes.

In the manufacturing process, equipment failure can be a threat to productivity. Therefore, it is important to build a systematic predictive maintenance model for process equipment from the perspective of preserving the product production schedule and quality, as well as from the perspective of preventing energy waste and accidents. For this purpose, we aim to identify various sensor data collected from the facility, analyze the comparison with the existing normal state, and build a predictive model for the future state of the facility. In this study, we use time series data obtained from sensors on pumps in a manufacturing process to cooperatively learn a 1DCNN-Bilstm model and evaluate the performance of a federated learning framework that considers the data distribution shifts of each machine. The 1DCNN-Bilstm model for time series anomaly detection and predictive maintenance is effective in extracting features from time series data and detecting anomalies, and effective predictive maintenance is possible through a federated learning framework that considers shifts in the distribution of time series data. In our experiments, the proposed framework achieved a test accuracy of 97.2%. We demonstrate the superior performance of the proposed framework compared to existing predictive conservation federated learning. The main contributions are as follows:

1. Proposal of a federated learning framework that considers time series data distribution shifts.
2. Proposal of a 1DCNN-Bilstm model for time series anomaly detection and predictive maintenance of manufacturing processes.

The rest of this article is organized as follows: Section 2 provides an overview of the background and related research in manufacturing process data and predictive maintenance, anomaly detection, and FL. Section 3 describes the proposed 1DCNN-Bilstm model for anomaly detection and predictive maintenance and the design of federated learning considering time series data distribution shifts. Section 4 presents and analyzes the evaluation results. Section 5 concludes the paper.

## 2. Related Work

### 2.1. Manufacturing Processes

Pumping equipment is responsible for moving fluids and is used in a variety of industries. Pumps are mechanical devices that transfer liquids or gases at a specific pressure or move them to a desired location. They play an important role in the manufacturing process and can fail for a number of reasons.

The importance of pump equipment is as follows:

- Fluid transfer: Pumps are responsible for transferring liquids or gases to a desired location. They are essential for accurately and efficiently moving a variety of fluids, such as raw materials, byproducts, and solvents, in manufacturing processes.
- Pressure maintenance: Some processes require a constant specific pressure. Pumps keep liquids or gases at a specific pressure, which allows stable and efficient processes.
- Mixing and separation: Some processes require mixing or separating multiple fluids. Pumps can perform these tasks, mixing fluids in the correct proportions or separating the desired components.

The causes of failure and the associated issues vary but, in general, involve the following:

- Mechanical failure: When parts inside a pump wear out or break, it can cause the pump to perform poorly or stop. Examples include worn shafts and bearings, leaking seals, and broken rotors or inverters.
- Fluid-related issues: Impurities, solid particles, and corrosion from the fluid handled by the pump can build up inside the pump and affect its performance. This can impede fluid flow or damage components.
- Changes in operating conditions: Such changes can put stress on the pump. For example, fluctuations in the temperature, pressure, and flow rate of the fluid can overload the pump or make it unsuitable for the operating conditions.

These failure causes and issues can be mitigated through preventive and regular maintenance. Preventive maintenance involves regular assessment of the condition of the pump to help prevent malfunctions. It is also important to implement a proper fluid management and filtering system to maintain the cleanliness and quality of the fluid [11,12].

### 2.2. Predictive Maintenance

**Figure 1:**
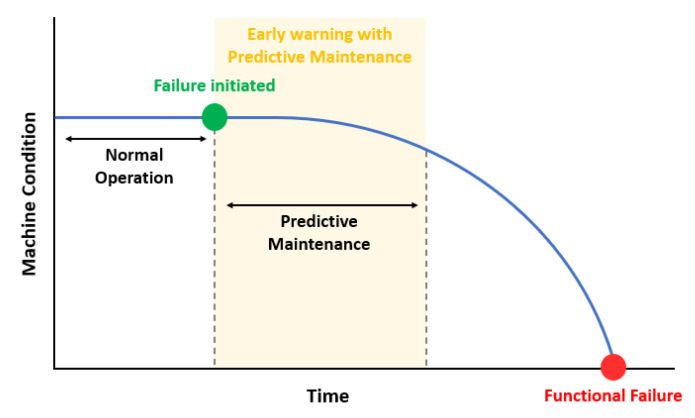
Predictive maintenance over time and equipment conditions.

Predictive maintenance (PdM) is a recent preventive maintenance approach to improve the performance and efficiency of manufacturing processes by increasing the life of equipment and ensuring sustainable operational management [13]. This results in reduced downtime, a lower number of unnecessary line stops, and reduced repair costs. It is important to maintain the highest efficiency and optimal condition of manufacturing equipment [14]. Methods are needed to ensure the efficiency of the equipment by minimizing machine downtime and preventing incidents in advance [13]. Since PdM is based on data collected from multiple sensors, it must address a variety of issues such as development cost, operations, and privacy [15]. To understand PdM, we need to define maintenance, which is the combination of all the techniques that allow a system to restore and maintain its ability to perform its required functions. As a simple example, Figure 1 is a potential failure diagram for PdM that takes into account time and the condition of the equipment [16]. Maintenance can be divided into four types. The first is simple replacement of parts when they fail. The second is scheduled periodic replacement of each part based on the failure history, which has the advantage of increasing the overall time efficiency of scheduled maintenance. However, periodic replacement of non-functional equipment is a waste of resources. The third method is proactive maintenance, which involves eliminating defective elements to improve the efficiency of the facility. The final method analyzes equipment data in attempt to identify and address abnormalities prior to failure. By solving problems such as periodic replacement and inaccurate analysis, it has the advantages of reducing the reliability and cost of facilities and extending their lifespan. Predictive maintenance is one of the core technologies of smart factories and is a technology that recognizes and predicts the causes and conditions of equipment failure and defects based on collected data for efficient management [17].

Predictive maintenance uses technological advances of real-time sensing and prediction algorithms related to the state of physical systems [18]. Though one of the most common keywords in Industry 4.0 is PdM [19], such systems are not widespread. One of the reasons for this is the diversity of data, the characteristics of which can vary even in the same work environment, making it difficult to reuse PdM models. In addition, data from industrial sites are time-dependent series data that require accurate preprocessing and feature extraction. The biggest problem is the imbalance of collected data, and it is difficult to secure equipment failure data. This hinders high performance during the training process, and various unsupervised learning methods are being researched to resolve the imbalance [20]. Predictive maintenance is advantageous over preventive maintenance and can reduce costs and downtime. However, many PdM systems are prohibitively expensive due to the cost of sensor and infrastructure installation, management of data storage and servers, and deployment of software. However, in recent years, many technologies and companies have aimed to improve machine learning capabilities and reduce sensor and infrastructure costs. Smart manufacturing technologies for Industry 4.0 production methods are accelerating with data-driven approaches. Machine vision-based PdM is a technology that monitors the status of machines and improves performance through diagnosis and detection of problems before they occur. This method offers several options such as vibration analysis and thermal imaging [21]. Research is also underway to use a managed digital twin of a turbine engine to illustrate the design of a model architecture. A previous study combined digital twin technology to predict the maintenance schedule of a machine to reduce costs and increase machine lifetime. Our aim is to extend experimentation to performance evaluation [22].

### 2.3. Anomaly Detection

**Figure 2:**
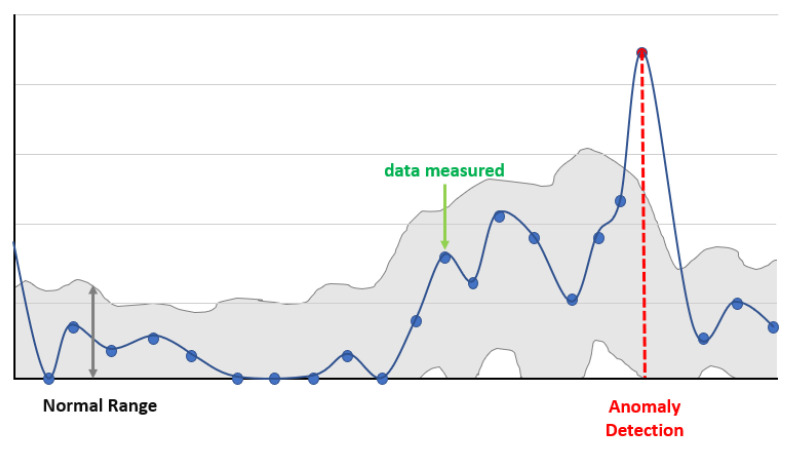
Monitoring anomaly detection in time series data.

An anomaly is defined as atypical data, such as that generated by an unusual pattern or data with a low probability density. Anomalies are expressed in various terms, and the definitions vary depending on the field or problem. Thus, there is no specific methodology, and the chosen method is based on the defined problem. Anomaly detection aims to detect abnormal patterns or anomalies to identify differences from normal conditions (Figure 2). Anomaly detection, the process of detecting abnormal conditions in a dataset, is an important area of analysis in signal research. Several approaches can be considered for defining anomalies, especially for time series sensor data. These approaches can be grouped into six main methodologies: statistical and probabilistic methods, pattern matching methods, clustering method, distance-based methods, predictive methods, and ensemble methods [23]. Most of the data used in the industrial field are time series data, and statistical research on anomaly detection using time series data has been conducted for a long time, most commonly using methodologies that consider the mean and variance over time. To achieve good performance and cost reduction with time series data, anomaly detection models need to be designed based on the context [24]. With such data, anomalies occur at a specific point in time. Most methodologies are based on Long Short-Term Memory (LSTM) or Convolutional Neural Networks (CNNs).

Anomaly detection in time series data collected from systems can be used to monitor system status and predict problems to avoid failure and reduce cost [25]. Various anomaly detection studies are ongoing. One study based on unsupervised learning using Transformer showed better performance than LSTM and CNNs in learning dynamic patterns of sequential data based on self-attention. The method consists of an encoder comprised of a Transformer encoder layer and a decoder containing a Conv1D layer [26]. It is difficult to obtain labeled data in anomaly detection research based on sequential data because anomalies are rare. Current anomaly detection methods are typically based on unsupervised or semi-supervised training, where sequences that deviate from normal patterns can be categorized as anomalies [27,28,29]. However, although these approaches can make predictions about sequences, they cannot explain the results, limiting their wide application [30]. Anomaly detection is difficult for a number of reasons, but the main one is lack of domain knowledge. The ‘normal’ pattern must be determined to then identify data as abnormal. In everyday situations, anomalous data are rare and often not obvious. Therefore, without established domain knowledge, it can be problematic to determine what is normal. This method also requires an understanding of the data being measured and generated, i.e., preprocessing domain knowledge. The second problem with anomaly detection is that training with limited amounts of anomalous and normal data can lead to poor performance. Therefore, unsupervised learning has been the main focus of research in anomaly detection in most domains, including system operation, security-related systems, and process data management. Anomaly detection and outliers are also used in business.

### 2.4. Federated Learning

**Figure 3:**
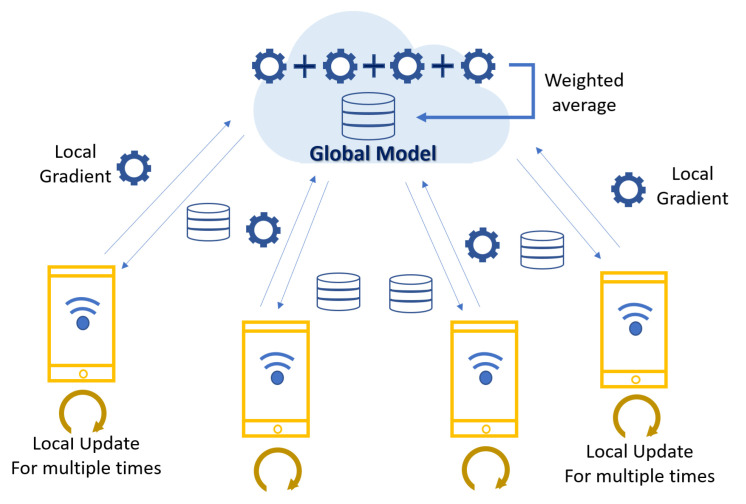
Illustration of FL framework proposed by Google.

Federated learning is a technology in which multiple local clients and a central server cooperate to learn a global model in a situation where data are decentralized, and local clients are IoT devices, smartphones, etc. After the first study [31] on such learning was published in 2015, it was introduced on Google’s blog [32] in 2017, and is starting to receive attention as a technology for KEYBOARD application. Federated learning enables learning without issues with data privacy and leakage. Most DL/ML models learn based on big data, which can cause privacy and security issues. Federated learning stores the data on each individual’s local client rather than on a centralized server, trains locally, and updates a model on a centralized server with the parameters of the updated model (Figure 3) [33]. The goal is to avoid data conflicts while protecting privacy. Multiple clients coordinate with one or more central servers for a distributed machine learning setup. When network traffic and costs increase during the training process, federated learning can reduce costs by only receiving updates from local models. The basic idea behind federated learning is that the central server modifies the global model with local updates. However, the central server is a single point of failure, which can be problematic. In federated learning, local devices or communities collaborate to learn. In comparison, distributed learning involves model training in an environment where data are stored on multiple servers.

Federated learning is essentially a distributed learning method in which multiple distributed clients individually train a machine learning model using their own local data under the coordination of a central server, which then aggregates the trained models into a final global model. Training is an iterative process of updating local models and aggregating the global model until the models converge or reach a predefined number of interactions. Federated learning represents a significant shift from centralized and expensive machine learning to a distributed approach that can use many computing resources [34]. In many networking applications, local learning is becoming necessary because large amounts of data are generated or collected at the network edge and cannot all be sent to the cloud due to issues such as network capacity constraints, latency requirements, and privacy concerns [35]. In its basic form, federated learning follows an iterative procedure to train a global model for use by all clients. A central server selects a subset of clients with whom to share the current global model, and each selected client updates the model using only local data and forwards the updated model to the central server, which aggregates the updated local models from the clients to update the global model. This process is repeated until certain convergence criteria are met [36]. The learning process of federated learning is as follows: calculate the weight values of the local data of each client, aggregate the weights of multiple local datasets, and convert the data to a shared global model. As federated learning is based on multiple devices and clients, the distribution of the population cannot be assessed with any local data. Also, the number of data points varies among individuals. Currently, federated learning is most actively researched in the medical field. As such data are privacy-sensitive, AI models must be developed in situations that prevent data leakage. In the existing architecture proposed by Google, individual mobile devices are connected to medical institutions, AI model training is performed using data from within the medical institution, and the learned parameters are sent to the central server to develop a global model. There are two main methods of federated learning, Federated Stochastic Gradient Descent (FedSGD) and Federated Averaging (FedAVG). Compared to FedSGD, which updates the parameters at every transmission, FedAVG transmits updated parameters from the mobile device, which reduces network costs and enables efficient use of mobile devices. In a related study, the results of the two methods were not significantly different, so FedAVG is recommended [8]. In addition to the medical field, there is active research on applying federated learning to communication networks. A previous study applied federated learning to edge networks to help determine the amount of data to offload from edge servers to cloud servers through multi-access edge computing [37]. An algorithm called BlockFL, which aggregates the preferences of multiple users and sends them to the edge server, is also being researched to cross-validate weights from users in combination with BlockChain for security.

## 3. Federated Learning for Predictive Maintenance and Anomaly Detection

In manufacturing, equipment failure can be a threat to productivity. Therefore, it is important to build a systematic PdM model for process equipment from the perspective of preserving product production schedules and quality, as well as from the perspective of preventing energy waste and accidents. For this purpose, we identify sensor data collected from the facility, compare the data with the existing normal state, and build a predictive model for the future state of the facility.

### 3.1. Overall Architecture

**Figure 4:**
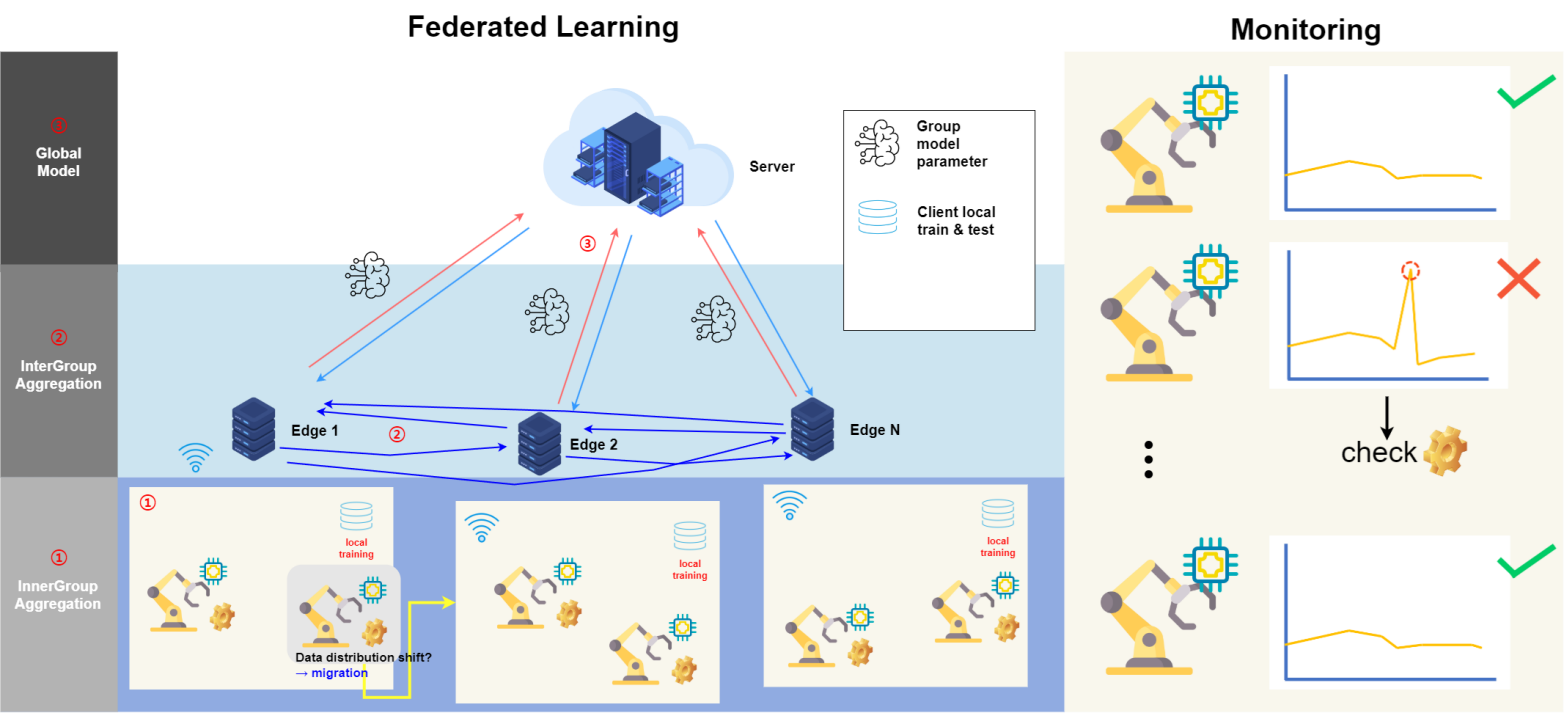
Proposed framework structure.

The proposed framework is a 1DCNN-Bilstm model for PdM series data distribution shifts in manufacturing. Figure 4 is a schematic illustration of the proposed framework, mainly composed of a client, a group (edge computing server), and a secondary server (cloud computing server); devices can exchange information through real-time communication. First, clients are mobile or IoT devices that collect real-time data from manufacturing equipment, and each client has an individual dataset. A group is a set of clients selected by the secondary server, and a group can be empty. A group creates a model by associatively learning the included clients. The secondary server is responsible for assigning clients to each group and aggregating the models generated by each group to create a common model. Unlike traditional federated learning frameworks, our model considers the shifting data distribution of each client and reassigns the client groups when a new device joins.

The model used by each client, group, and secondary server is 1DCNN-Bilstm, which is proposed for anomaly detection in time series data. This model consists of a Conv1D-based feature fusion layer, an LSTM-based time series prediction layer, and an output layer. The input layer contains sliding window processed data. Each variable is weighted by the CNN, and the information between variables is combined to predict time series anomalies.

The detailed roles of clients, groups, and secondary servers in the framework are as follows:

- Client:
  Each client has its own local dataset, which consists of data collected in a specific environment. The client trains its local model using its own dataset. The training is customized to the client’s environment, and the trained model is used for the client’s updater. Updates to the trained local model are sent to the group or secondary server. The updates contribute to the model global update through intra-group and inter-group aggregation.
- Groups:
  Grouping clusters clients into groups with similar characteristics. This allows similar model updates within the group. The model updates of the clients in the group are aggregated to train the group model. This takes into account the characteristics of the clients in the group, and the group model is used to initialize client updates. This model aggregates the updaters of the clients in the group to form a global model update.
- Auxiliary Server:
  The auxiliary server provides the group with an initial model and optimization di- rection and serves as a starting point for the group to begin training its local model. This server receives aggregated updates from within the group, performs inter-group aggregation, and combines updates from group models to form a global model of the entire system. The auxiliary server designs an algorithm for cold-starting new participants and to determine how new clients are assigned to groups to start learning.

**Figure 5:**
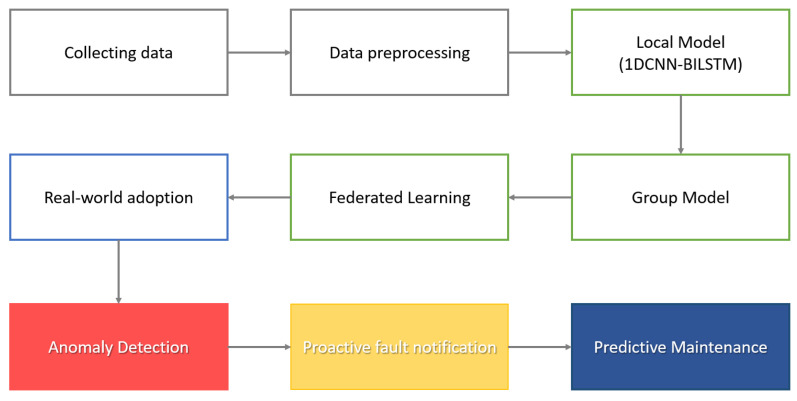
Suggested predictive maintenance process.

Figure 5 shows the predictive maintenance process using federated learning. First, data are collected for each facility in real time through sensors or recording logs. Then, the raw data are analyzed and preprocessed to increase anomaly detection, improving the quality of the data to extract useful information. Data preprocessing improves the quality of the data, removes noise or missing values, extracts useful features, and formats the data. The preprocessed data are divided by device or group to perform federated learning. Federated learning is used to build models while protecting data between individuals or organizations in a distributed data environment. Each client (device or group) trains a model using local data, shares information on a central server, and combines the models to create an improved global model. The predictive maintenance model generated through federated learning is applied to the actual manufacturing process, and data from equipment are collected in real time and analyzed using PdM models. The model detects patterns in the data based on anomaly detection algorithms and sends notifications to field managers when there is an anomaly in the signal. After receiving the notification, the field manager can inspect the equipment and take necessary actions. This allows rapid identification and resolution of equipment anomalies, ensuring efficient use and maintenance of equipment. This process can be used to build a PdM system with data preprocessing and federated learning to analyze data in real time and increase efficiency and reliability in detecting and acting on abnormalities.

Thus, clients use local data to train a local model, 1DCNN-BiLSTM, and send updates to the model, while groups aggregate updates from clients in the group to form a group model and perform inter-group aggregation. The auxiliary server provides the initial model and optimization direction, performs inter-group aggregation, and handles the cold start of new participants. This process refers to the procedure when a single round of association learning is performed, and the goal is to improve the accuracy of the model by repeating the process multiple times. In other words, it represents a single process from the client to the joint anomaly detection model. This allows efficient model updates and participant management in a federated learning system, enabling efficient anomaly detection.

### 3.2. Time Series Data Distribution Shifts

The federated learning technique adopted in this paper performs federated learning by considering the distributional shifts of clients [34]. Data distribution shifting is a natural phenomenon in training devices that mainly use IoT nodes such as industrial sensors, wearable devices, and cameras. These IoT nodes collect data from the environment and are often used for training, where the data distribution may change over time. This change in data distribution can affect the performance of the model, so a flexible data distribution strategy is needed.

Static clustering methods are used to initially divide data into groups of clients. However, as data distribution shifts, this clustering structure can be difficult to maintain. Therefore, we introduce a flexible data distribution strategy to solve this problem. The flexible migration strategy uses the Wasserstein Distance to detect training data distribution shifts for all clients (except cold clients) before each training round. If the Wasserstein Distance exceeds a predefined threshold i, that client schedules a new training start phase, called a cold start. This allows us to identify clients whose data distribution has changed significantly and to treat them as appropriate.

The formula below is defined based on the Wasserstein Distance with a distribution shift threshold i for client ci. The Wasserstein Distance is used as a metric to measure the distance between two probability distributions. If this distance exceeds the preset threshold i, the client’s data distribution shift is large, and a cold start is scheduled. This allows the identification of clients that are sensitive to data distribution shifts and treatment to improve the performance of the model.

(1) $$\tau_{i}=\frac{0.2}{LabelSize}n_{i},$$

A cold start algorithm is used to ensure that clients within each group have an initially fair start. The algorithm detects shifts in the distribution of training data among clients and aims to exclude clients with large early shifts using the distance measure EDC (Euclidean distance of Decomposed Cosine similarity). The EDC decomposes a client’s model updates in a certain direction and computes the similarity between the update direction and the specific direction to measure the similarity between clients. The EDC formula is defined as follows [34]:

(2) $$EDC\left(i,j\right)=\frac{1}{m}\sqrt{\sum_{v\in V}{\left(S(i,v)-S(j,v)\right)}^{2}},$$

(3) $$EDC\left(i,j\right)=\frac{1}{m}∥K\left(\Delta w_{t}^{\left(c_{i}\right)},V^{T}\right)-K\left(\Delta w_{t}^{\left(c_{j}\right)},V^{T}\right)∥,$$

(4) $$V=SVD\left(\Delta W^{T},m\right),V\subset R^{d_{w}\times m}.$$

where *V* is the matrix obtained after decomposing the model updates in a specific direction, computed using Singular Value Decomposition (SVD). The matrix *V* is $R^{d_{w}\times m}$ in dimension.

When EDC is used to calculate the distance between clients, those with large distributional shifts are far distant from other clients; those above a distance threshold are scheduled for a cold start. This eliminates clients with large early distribution shifts and allows grouped clients to achieve stable performance. Thus, the cold start algorithm mitigates the performance degradation caused by an initially unbalanced data distribution and allows grouped clients to train more consistently.

### 3.3. Anomaly Detection Model

**Figure 6:**
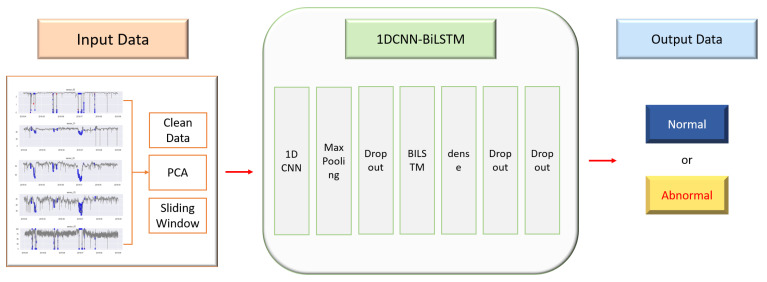
Proposed 1DCNN-BiLSTM anomaly detection model.

The 1DCNN-BiLSTM is a model proposed for time series anomaly detection and PdM. The preprocessed data are converted to the input form of the BiLSTM using the sliding window technique and are used as the input of the model. Second, a CNN is used to extract features from the time series data. Third, the BiLSTM is used to predict anomalies in the feature-extracted data. Figure 6 shows the process of anomaly detection.

1. Data preprocessing
  For data preprocessing, the relationship between variables is assessed through correlation analysis of data columns. Then, outliers and missing values in the data are detected and removed or replaced. PCA is used to reduce the dimensionality of the data by extracting principal components considering the correlation between variables. Since Bi-LSTM has a three-dimensional shape in the form of samples, time steps, and features, it converts the data into a sequence through a sliding window. In the sliding window, the step is set to 1/2 of the window size to reduce duplicate data.
2. 1DCNN-BiLSTM
  The layers of the model consist of a 1D convolution layer, maxpooling, dropout, two Bi-LSTM layers, and two hidden layers.
3. 1DCNN
  To extract fine-grained features of a time series, we use a one-dimensional convolutional neural network (1D CNN) on the input data using a filter. The resulting value is calculated as an output value through an activation function that uses the Rectified Linear Unit (ReLU) function. Through this process, local features of the input data are extracted and used to learn global features. After that, the size of the output value is reduced in the maxpooling layer, and the performance is improved by preventing overfitting in the dropout layer.
4. BiLSTM
  Bidirectional LSTM is used to detect anomalies in time series data. BiLSTM processes the input sequence in both directions, allowing it to take into account both the information before and after the sequence. The BiLSTM computes the input sequence $x=(x_{1},x_{2},\cdots ,x_{n})$ as a forward hidden sequence $\vec{h_{t}}=\left(\vec{h_{1}},\vec{h_{2}},\cdots ,\vec{h_{n}}\right)$ and a backward hidden sequence $\overset{\leftarrow }{h_{t}}=\left(\overset{\leftarrow }{h_{1}},\overset{\leftarrow }{h_{2}},\cdots ,\overset{\leftarrow }{h_{n}}\right)$ in opposite directions, with each LSTM sharing equal weights in the forward and backward propagation process. The encoded vector is formed by concatenation of the final forward and backward outputs, $y_{t}=\left[\vec{h_{t}},\overset{\leftarrow }{h_{t}}\right]$, where ht is a vector of size 2 X h, and h is the hidden state size of the LSTM [38].
  (5) $${\vec{h}}_{t}=\sigma \left(W_{\vec{h}x}x_{t}+W_{\vec{h}\vec{h}}{\vec{h}}_{t-1}+b_{\vec{h}}\right),$$
  (6) $${\overset{\leftarrow }{h}}_{t}=\sigma \left(W_{\vec{h}x}x_{t}+W_{\vec{h}\vec{h}}{\overset{\leftarrow }{h}}_{t-1}+b_{\vec{h}}\right),$$
  (7) $$y_{t}=W_{y\vec{h}}\vec{h_{t}}+W_{y\vec{h}}\overset{\leftarrow }{h_{t}}+b_{y}$$
  The output from Bilstm was processed through a dense layer and a dropout layer, and the final output was used for anomaly detection using the softmax function.

## 4. Experiment and Results

### 4.1. Experiment Environments

Table 1 shows the experimental environment of the hardware and software used to conduct the study. The hardware configuration includes an AMD Ryzen 7 6800HS processor and an NVIDIA GeForce RTX 3060 graphics card. The operating system used in the software is Windows. As a programming language, we used Python 3.7, which is widely used in the field of data analysis and machine learning. We also utilized TensorFlow 2.11.0, an open-source machine learning framework, to develop and run our model. TensorFlow provides a variety of libraries related to deep learning model development.

### 4.2. Dataset

#### 4.2.1. Data Definition and Introduction

This study uses pump sensor data for predictive maintenance, a public dataset from Kaggle. The data collection period was from 1 April 2018 to 31 August 2018, and the total number of data points was 220,320. The data were collected through 51 sensors with time stamps and was assigned one of three labels: ‘NORMAL’, ‘BROKEN’, or ‘RECOVERING’.

#### 4.2.2. Data Preprocessing

**Figure 7:**
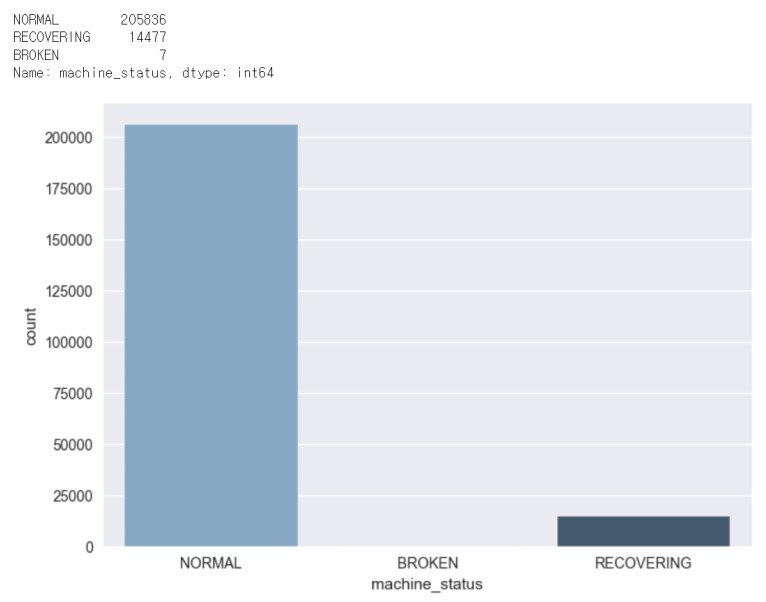
Comparison of the number of data classes.

As shown in Figure 7, the data is unbalanced with a large difference in the number of data in each class. In the figure, there are 205836 ‘NORMAL’ data, 4477 ‘RECOVERING’ data, and 7 ‘BROKEN’ data. Since the number of ‘RECOVERING’ and ‘BROKEN’ classes is small compared to the number of ‘NORMAL’ classes, we combined ‘RECOVERING’ and ‘BROKEN’ into one class (anomaly).

**Figure 8:**
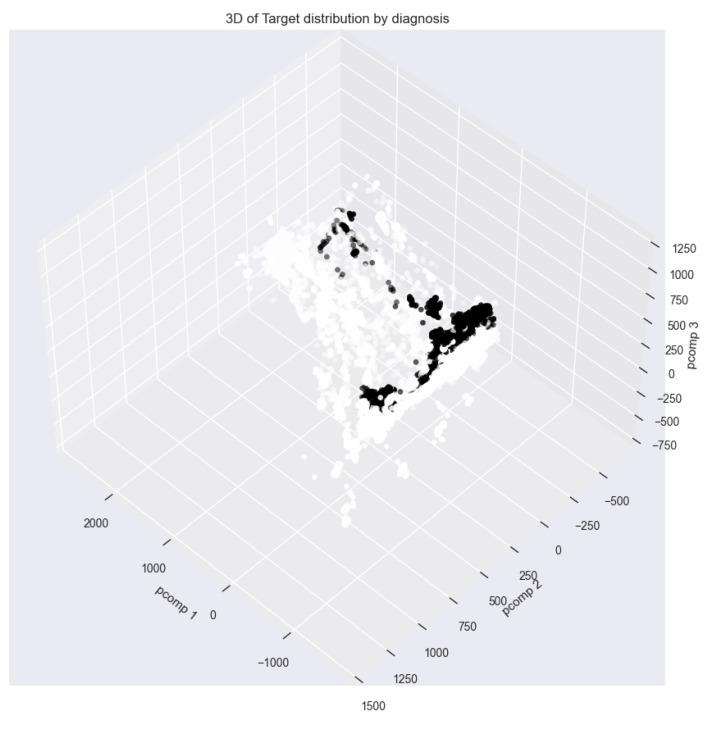
Visualization of PCA results.

We also used PCA to reduce the characteristics of the data (Figure 8). Principal component analysis revealed that 73.5% of the variance in the original dataset lies on the first principal axis, 8.3% on the second principal axis, and 3.6% on the third principal axis. This reduces the dimensionality of the data from 52 to 3.

#### 4.2.3. Data Separation

The training and test datasets represented 75% and 25% of the total data, respectively. To allocate the data to all clients in the federated learning, we varied the number of data points among clients to take advantage of the time series nature of the data. We set the total number of clients to 30.

#### 4.2.4. Learning Path for Predictive Maintenance

The separated data is assigned to each client for association learning, and each client is randomly assigned to a group. Within the group, each client trains a BI-LSTM model to create a model that can classify the pump data as normal or abnormal, and the weights of each client’s model are aggregated to create a group model. After that, the weights of each client’s model are aggregated between groups, and a joint model is created to perform association learning.

### 4.3. Performance Metrics

#### 4.3.1. Weighted Accuracy

The experiments in this study were evaluated using weighted accuracy, a metric that measures the performance of a model considering the imbalance in the data distribution. The weighted accuracy used in CFL-based frameworks is calculated by applying weights proportional to the size of the test dataset in each group and is expressed by the following formula:

(8) $$WeightedAccuracy=\frac{\sum_{i=1}^{C}\left(Accuracy_{i}\times Weight_{i}\right)}{\sum_{i=1}^{C}\left(Weight_{i}\right)}$$

The “weighted sum of the number of correctly classified samples” is calculated considering the numbers of misclassified samples in each group based on test dataset size. The weighted accuracy depends on the test dataset size of each group, and the overall performance can be evaluated by considering the imbalance of the data distribution. This ensures that the difference in performance among groups is taken into account, and that the performance of the group with the larger dataset has a greater impact on the overall performance. Using weighted accuracy, the performance of a model can be evaluated fairly, and the bias caused by an imbalance in the data distribution can be mitigated.

#### 4.3.2. Data Discrepancy

Mismatch indicates the degree of difference in model parameters between the global model and the clients, with lower values indicating greater agreement between the models. Below is the formula for dissimilarity [34].

(9) $$Discrepancy\left(t\right)≜\frac{1}{|S_{t}|}\sum_{i\in S_{t}}∥w_{i}-w_{t}∥.$$

For every client *i* in the client set $S_{t}$, the Euclidean distance between that client’s model parameters $w_{i}$ and the global model parameters $w_{t}$ is computed and averaged, as a formula for the discrepancy at time *t*. The goal of federated learning is to minimize the dissimilarity, and strategies can be used to achieve this, such as coordinating model updates between clients or using a weighted average to update the global model. The dissimilarity is calculated as the value at time *t* and can be used to observe the change in dissimilarity over time as federated training progresses and to understand the progress of model training.

### 4.4. Results

In this paper, we consider client-level distributional shifts. FlexCFL uses an approach that updates the global model by aggregating updates from grouped clients. Each client performs an update within its group, which is combined with updates from other clients in the group. The combined updates are treated with the same weight as updates from other clients in the group. A portion of the updates that a client produces during training are aggregated with the updates from other clients in the group and contribute to the update of the global model. This allows FlexCFL to combine the knowledge of grouped clients to build a stronger and more efficient global model. By playing an important role in coordinating updates from grouped clients and managing the variance of model training, FlexCFL can improve the performance of federated learning while balancing the differences between client groups. For our experiments, we compared the performance of 1DCNN-BILSTM, 1DCNN-LSTM, and LSTM models in combination with the federated learning frameworks FedAVG, FlexCFL, FeSEM, and IFCA. Each framework and model is compared in terms of test accuracy, train loss, and discrepancy.

The following is a description of the federated learning framework used in our experiments:

- FedAvg: Vanilla FL framework. A federated learning framework that performs local training of clients in parallel and averages the central model [31].
- IFCA: A framework that optimizes model parameters for user clusters via gradient descent while estimating the user’s cluster ID [39].
- FeSEM: A distance-based CFL framework that probabilistically minimizes the expected disagreement between *ℓ*2 clients and groups [40].

For our experiments, each framework performed a total of 100 rounds of association learning. At the end of each round, we derive the accuracy of the global model on test data for performance evaluation. Finally, we select the largest accuracy among the derived accuracies for each round and consider it as the final performance of each framework.

#### 4.4.1. Test Accuracy

**Figure 9:**
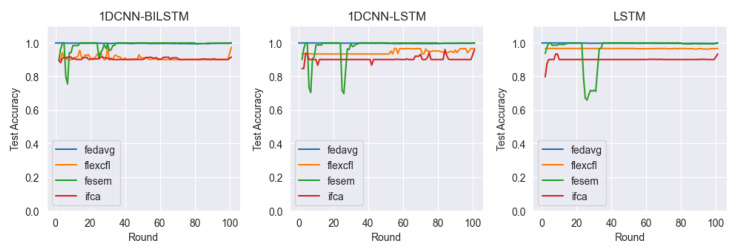
Train Accuracy.

Figure 9 and Table 2 compare the test accuracy of each framework and model. The experimental results show that FlexCFL-1D-BILSTM achieves 97.2%, FlexCFL-1D-LSTM performs 96.5%, and FlexCFL-LSTM performs 96.6% in terms of accuracy. Within the FlexCFL framework, we can see that 1DCNN-BILSTM has the highest accuracy. Among the federated learning frameworks, FedAVG and FeSEM have the highest accuracies in the 99% range, while IFCA has the lowest accuracy. However, we can see that the overall accuracy of the models is between 91% and 99%. For FeSEM, there seems to be a lot of variation in the initial accuracy across the three models.

#### 4.4.2. Train Loss

**Figure 10:**
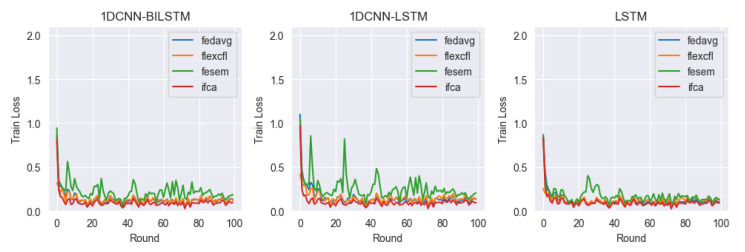
Train Loss.

Figure 10 shows a visualization of the train loss of each framework versus the model. We can see from the train loss that the models are generally well trained, except for FeSEM. In the case of FeSEM, we can see that the train loss is somewhat deviated, suggesting that the model has not yet learned enough and has reached a local minimum, or that there is insufficient training for certain classes due to data imbalance. The LSTM tends to have the most stable decrease in train loss, while the 1DCNN-LSTM is the most unstable.

#### 4.4.3. Discrepancy

**Figure 11:**
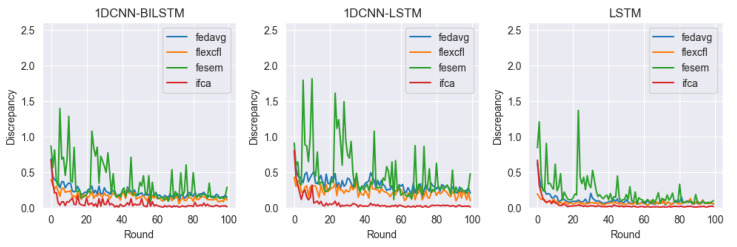
Discrepancy.

Figure 11 shows a visualization comparing the discrepancy of the models with each framework. From the experimental results, we can see that FeSEM tends to have a very high variance of discrepancy values for all three models, which indicates that the models are likely overdispersed. We can see that FeAVG has the lowest discrepancy among all three models. Overall, 1DCNN-LSTM tends to have the largest deviation from the discrepancy value, and LSTM has the lowest.

Until now, we have compared each framework and model combination through three metrics: test accuracy, train loss, and discrepancy. When the proposed model is a combination of Bi-LSTM and a federated learning framework, FedAVG, FeSEM, FlexCFL, and IFCA perform well in the order of test accuracy, but when other metrics are also considered, FeSEM does not learn the model well. Therefore, except for FeSEM, we can see that FeAVG performs the best in test accuracy, train loss, and discrepancy, and FlexCFL performs the second best. In the case of IFCA, we can see that it has the lowest performance among the compared frameworks, but it is not much worse than the performance of the other frameworks.

## 5. Conclusions

This paper emphasizes the importance of PdM in manufacturing processes and proposes a federated learning framework and a 1DCNN-Bilstm model that considers time series data distribution shifts. In this paper, a combination of 1DCNN-BILSTM, 1DCNN-LSTM, and LSTM models and federated learning frameworks FedAVG, FlexCFL, FeSEM, and IFCA are used for time series data preprocessing and experimentation, and each framework and model are compared in terms of test accuracy, train loss, and discrepancy. The experimental results show that the proposed framework achieves 97.2% test accuracy. Although the proposed structure did not show the best performance when compared to other frameworks and model combinations, it can be said that the proposed framework has the second best performance after FedAVG because the deviation of the test accuracy and training loss of the model in each round of FeSEM is large and it is judged that proper learning is not achieved.

In future research, we plan to develop this work to monitor the condition of the equipment to enable real-time predictive maintenance. In addition, we plan to develop data preprocessing and compression techniques to reduce the amount of data transmission in the edge computing structure by visualizing the data collected for predictive maintenance so that managers can understand at what point a problem occurs. We will convert the data collected from edge devices into the required information and utilize data compression algorithms to realize efficient data transmission. By carrying out the above research plan, it is expected that a real-time predictive maintenance system utilizing a distributed edge computing system can be built more efficiently.

## Figures and Tables

**Table 1:** System specification.

Hardware Environment	Software Environment
CPU: AMD Ryzen 7 6800HS	Window 11, Tensorflow 2.11.0
GPU: NVIDIA GeForce RTX 3060	Python 3.7

**Table 2:** Test Accuracy: using a combination of anomaly detection models LSTM, 1D-LSTM, and 1D-BILSTM and federated learning FedAVG, FeSEM, IFCA, and FlexCFL to measure test performance.

Model	FedAVG	FeSEM	IFCA	FlexCFL
1D-BILSTM	0.998571455	0.998552315	0.914563614	0.972923049
1D-LSTM	0.998571455	0.998552315	0.959681333	0.965874879
LSTM	0.998571455	0.998552315	0.933049078	0.966081691

## Data Availability

Pump sensor data for predictive maintenance (https://www.kaggle.com/datasets/nphantawee/pump-sensor-data) (accessed on 4 March 2019).
